# Electricity in the Extraction of Teeth

**Published:** 1859-04

**Authors:** Decatur P. Gregg

**Affiliations:** Greensboro, N. C.


### ARTICLE XII.

### *Electricity in the Extraction of Teeth.*

Messrs. Editors:

My object in addressing you is not for the purpose of giving you an elaborate essay on the use of electricity as applied to the extraction of teeth. Neither do I wish to enter into any argument respecting it, but to furnish you with my own experience, which I think is of more value than any theoretical effort I could make.

I can do this the more satisfactorily perhaps by furnishing you some of the details, as they took place, than I can in any other way. The experiments were brief, but quite full enough for my comfort. I have extracted about ten teeth *with* the use of the battery, and in no case could I determine whether I ought to commend its use, or throw it aside. In this dilemma, I concluded to be the *patient myself*—I had two upper centrals, one lateral, and one cuspid that I wished extracted—crowns, worn away by mechanical abrasion—nerves not exposed—abundance of secondary dentine—no periosteal inflammation—teeth not sensitive. Present Drs. H. & B.—two reliable operators ; the first being an expert in the use of the battery. By way of experiment, the lowest graduated current was applied to each tooth at different times, and regularly increased, and every time the current was applied it was not only disagreeable, but of the most *exquisitely painful* character. The operation was frequently repeated with *always* the same result. I do not complain at trifles. The pain was really so *agonizingly excessive* that the perspiration would start out from the forehead as visibly as if an instrument was piercing the nervous pulp, continuously, without it being cut off at the apex of the tooth. When the current was taken off the pain ceased.

These experiments being *very satisfactory* to me thus far, there remains but one step further for me to go, to get at *very definite* conclusions. The forceps was then placed upon one of the fangs, the current applied, and the tooth quickly extracted. Did you suffer any pain from the extraction? you inquire. The extraction gave *no pain*, but the application of the electrical current was a *great deal* more painful than any extraction I ever submitted to, and I claim to know something about it, as I have had twenty-four taken out at different periods.

I was persuaded to have continued the electrical experiments, and have the remaining three taken out, but I respectfully declined the "rack and torture" any longer. The others were extracted at the same sitting, without any of the "modern improvements," with comparatively little pain, although, their conditions were similar to the other, only those were more firmly inserted. I offer no one any of my speculations about the matter, but merely suggest that if any operator wishes to get at *very satisfactory* and certain information, he do as I did, "put a hornet in his ear to get out the fly."

DECATUR P. GREGG.

Greensboro, N. C., *January 24th,* 1859.

---

### SELECTED ARTICLES.

